# Polymer-Modified Single-Walled Carbon Nanotubes Affect Photosystem II Photochemistry, Intersystem Electron Transport Carriers and Photosystem I End Acceptors in Pea Plants

**DOI:** 10.3390/molecules26195958

**Published:** 2021-10-01

**Authors:** Nia Petrova, Momchil Paunov, Petar Petrov, Violeta Velikova, Vasilij Goltsev, Sashka Krumova

**Affiliations:** 1 Institute of Biophysics and Biomedical Engineering, Bulgarian Academy of Sciences, Acad. Georgi Bonchev Str., Bl. 21, 1113 Sofia, Bulgaria; sakrumo@gmail.com; 2 Faculty of Biology, Sofia University ‘St. Kliment Ohridski’, 8 Dragan Tsankov Blvd., 1164 Sofia, Bulgaria; mokavey@abv.bg (M.P.); goltsev@gmail.com (V.G.); 3 Institute of Polymers, Bulgarian Academy of Sciences, Acad. Georgi Bonchev Str., Bl. 103-A, 1113 Sofia, Bulgaria; ppetrov@polymer.bas.bg; 4 Institute of Plant Physiology and Genetics, Bulgarian Academy of Sciences, Acad. Georgi Bonchev Str., Bl. 21, 1113 Sofia, Bulgaria

**Keywords:** single-walled carbon nanotubes, photosynthetic apparatus, JIP test, modulated reflection at 820 nm, chlorophyll fluorescence

## Abstract

Single-walled carbon nanotubes (SWCNT) have recently been attracting the attention of plant biologists as a prospective tool for modulation of photosynthesis in higher plants. However, the exact mode of action of SWCNT on the photosynthetic electron transport chain remains unknown. In this work, we examined the effect of foliar application of polymer-grafted SWCNT on the donor side of photosystem II, the intersystem electron transfer chain and the acceptor side of photosystem I. Analysis of the induction curves of chlorophyll fluorescence via JIP test and construction of differential curves revealed that SWCNT concentrations up to 100 mg/L did not affect the photosynthetic electron transport chain. SWCNT concentration of 300 mg/L had no effect on the photosystem II donor side but provoked inactivation of photosystem II reaction centres and slowed down the reduction of the plastoquinone pool and the photosystem I end acceptors. Changes in the modulated reflection at 820 nm, too, indicated slower re-reduction of photosystem I reaction centres in SWCNT-treated leaves. We conclude that SWCNT are likely to be able to divert electrons from the photosynthetic electron transport chain at the level of photosystem I end acceptors and plastoquinone pool in vivo. Further research is needed to unequivocally prove if the observed effects are due to specific interaction between SWCNT and the photosynthetic apparatus.

## 1. Introduction

In the recent decade, carbon-based nanomaterials and single-walled carbon nanotubes (SWCNT) in particular have been involved in the development of state-of-the-art approaches in agronomy and plant biotechnology. SWCNT were applied in the newly evolving field of plant-based nanobionics turning living plants into monitoring systems for nitroaromatic compounds [1]. Chitosan-complexed SWCNT were demonstrated to serve as a DNA carrier in a novel technique for genetic transformation of the chloroplast genome in a number of plant species [2]. The spectroscopic and electronic properties of SWCNT [3] intuitively make them highly appropriate candidate to be utilized in development of novel techniques for augmentation of photosynthesis and modulation of stress responses in photosynthetic organisms. Many of the predicted applications of SWCNT in plant biotechnologies require knowledge of their modes of action on the photosynthetic machinery. SWCNT were assumed to endow chloroplasts with wider photosynthetic action spectrum due to their absorbance in the ultraviolet, visible and near-infrared regions [4]. Giraldo et al. [4] also argued that composite nanoparticles consisting of semiconducting SWCNT and nanoceria are able to passively enter isolated higher plant chloroplasts and enhance photosystem II (PSII) activity and photosynthetic electron transport possibly by transferring excitons to the photosynthetic apparatus which might be employed as a beneficial strategy for increasing plant survival in shade conditions. In vitro studies of the interaction between photosystem I (PSI) and SWCNT point to electron transfer from PSI electron carriers F_A/B_ to SWCNT [5,6]. It is to be noted, however, that this effect was only observable at particular orientation between SWCNT and PSI complexes. Furthermore, Dorogi et al. [7] proved that SWCNT stabilize the charge separation in isolated purple bacterial reaction centres, thus providing another evidence for the possibility for direct interaction between SWCNT and components of the photosynthetic apparatus. 

SWCNT can penetrate the plant cell wall and cytoplasmic membrane and exert effects ranging from stimulation of cell growth (at low dose) to reactive oxygen species formation and necrosis (at high dose) in *Arabidopsis* mesophyll protoplasts [8]. Shen et al. [9] also, report on adverse effects of protoplast viability after treatment with SWCNT. SWCNT translocated to the chloroplasts are thought to cause significant changes in the thylakoid membrane’s architecture [8]. Indeed, the expression of genes related to chloroplast development was enhanced in rice seedlings treated with SWCNT [10]. Besides, the information gained from in vitro studies, relatively little is known on how SWCNT affect higher plants photosynthesis in vivo. While the chlorophyll (Chl) a and b content of rice seedlings grown from SWCNT-treated seeds did not change, the photosynthesis rate of these plants significantly increased [10]. Arabidopsis thaliana leaves infiltrated with SWCNT showed increased photosynthetic electron transport rate [4]. In addition to augmentation of photosynthesis, the action of SWCNT in intact plants was related to increased expression of antioxidant enzymes [10] which was found to alleviate the drought stress effects [11]. Our recent findings demonstrated that high doses of polymer-grafted SWCNT exert negative effects on the rate and efficiency of photosynthesis, when applied via foliar spraying [12]. We hypothesized that SWCNT interact with the components of electron transport chain. Extensive and detailed study of the Chl fluorescence of SWCNT-treated plants can help to answer the question of whether transfer of excitons and/or electrons from SWCNT towards photosynthetic complexes occurs in vivo and how it affects the efficiency of the photosynthetic process. JIP test of the prompt Chl *a* fluorescence related to the electron transport in PSII and analysis of the modulated reflection at 820 nm (MR) associated with the electron transport at the level of PSI are widely applied approaches in numerous works aimed at revealing not only the stress responses of the photosynthetic apparatus but also the general physiological condition of plants subjected to various types of stress [13,14,15,16,17,18]. 

In the current work we aimed to shed light on the putative interaction between SWCNT and the photosynthetic complexes in intact pea plants sprayed with aqueous solution of SWCNT grafted with ‘Pluronic’ P85 triblock co-polymer [19]. We found that, besides partial inactivation of PSII reaction centres, SWCNT also interfere with the photosynthetic electron transport chain at the level of intersystem electron carriers and the PSI acceptor side.

## 2. Results

### 2.1. Prompt Chlorophyll Fluorescence Induction Curves

**Figure 1:**
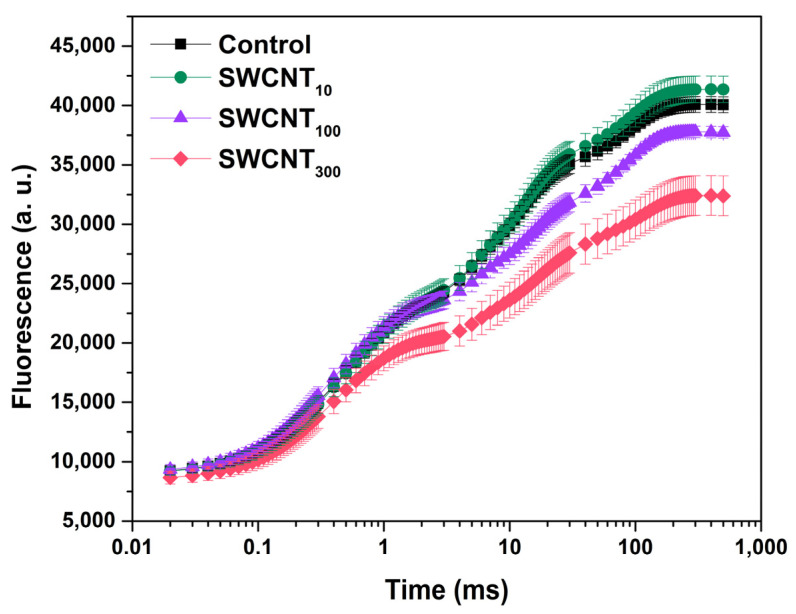
Prompt Chl fluorescence induction curves (±SEM) of intact pea leaves sprayed with distilled water (control), 10, 100 or 300 mg/L of polymer-grafted SWCNT.

The prompt Chl fluorescence transients of the control, as well as all treated plants, exhibited the characteristic points O, J, I and P (Figure 1). The fluorescence rise from O to J is related to the reduction state of Q_A_, with equilibration between the rates of Q_A_ reduction and re-oxidation being reached at J point [14,20]. Increase of fluorescence in the J-I phase is ascribed to gradual reduction of the PQ pool [21]; the fluorescence rise in the I point slows down due to reaching equilibrium between PQ reduction and re-oxidation; the P point is related to full reduction of the pool of PSI end electron acceptors [22]. Treatment with 10 mg/L nanotubes (SWCNT_10_) had little effect on both the shape and intensity of the prompt fluorescence curve. In the samples treated with 100 mg/L SWCNT (SWCNT_100_) we observed decrease of the fluorescence intensity after the J point and in leaves sprayed with 300 mg/L SWCNT (SWCNT_300_) an overall lowering of the prompt fluorescence intensity was visible (Figure 1). Notably, the latter effect was the most strongly expressed for P (maximal fluorescence, F_M_)—ca. 25% decrease in SWCNT_300_ relative to the control, while the fluorescence intensity at O was decreased by ca. 15%. The prompt fluorescence transients of pea plants treated only with the co-polymer in the corresponding concentrations, i.e., P85_10_, P85_100_ or P85_300_, were indistinguishable from the ones that are characteristic of the control plants (Figure S1A).

### 2.2. Differential Curves—Variable Chlorophyll Fluorescence Differences during J-I and I-P Induction Phases

**Figure 2:**
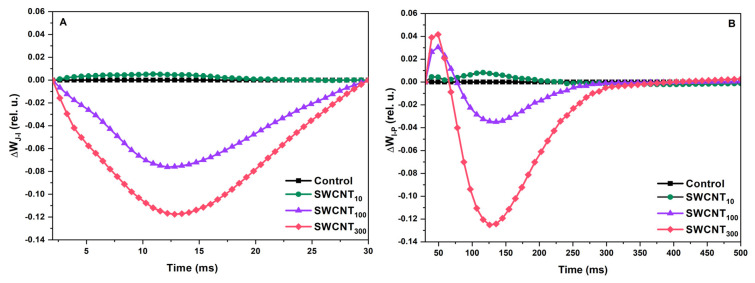
Differential curves constructed for the J-I (**A**) and I-P (**B**) phases of the prompt Chl fluorescence transients of pea plants sprayed with distilled water (control), 10, 100 and 300 mg/L SWCNT. The differential curves were obtained by subtraction of the control J-I and I-P phases from the respective ones of treated plants following normalization between the J and I or I and P points, respectively: ΔWJI = W_JI_^treated^–W_JI_^control^ and ΔW_IP_ = W_IP_^treated^–W_IP_^control^.

Detailed examination of the shape of the prompt Chl fluorescence curves was done by construction of differential curves. Treatments with the P85 polymer only, in any of the selected concentrations, did not induce significant alterations in the shape of the fluorescence induction curves (Figure S1). SWCNT_10_ did not strongly affect the shape of the fluorescence transients as seen from the resulting differential curves with values approximating 0. The differential curves constructed in the phase between the O and J points (ΔW_OJ_), known to bear information about the oxygen-evolving complex functionality [23], did not show significant deviation from the control for any of the tested SWCNT concentrations (data not shown). However, well reproducible negative bands in the differential curves in the J-I (ΔW_JI_, Figure 2A) and I-P (ΔW_IP_, Figure 2B) phases were found for the leaves treated with SWCNT_100_ and SWCNT_300_. These bands revealed significant changes in the shape of prompt Chl fluorescence transients in both variants relative to the control due to slower increase of the fluorescence intensity in the respective phases. This effect was concentration-dependent since it was manifested to a higher extent for the SWCNT_300_ concentration.

### 2.3. JIP Test 

To further substantiate our observations on the photosynthetic electron transport in SWCNT-treated plants we analysed the variable Chl fluorescence transients according to the mathematical expressions contained in the JIP test (Table S1) [14,17,20,24]. The fluxes of light energy which is absorbed (ABS/RC), trapped (TR_0_/RC, data not shown) and consequently utilized for electron transport (ET_0_/RC, data not shown) were not changed upon SWCNT_10_ (Table 1) and P85 (data not shown) application. None of the applied SWCNT concentrations affected the initial rate of active PSII reaction centres closure, M_0_ (Table 1). However, the turnover number of Q_A_ reduction/re-oxidation (N) increased along with SWCNT concentration reaching values higher by 32% in the SWCNT_300_ than in the control. The enhancement of N was correlated with similar increase in the S_m_ parameter. S_m_ corresponds to the area above the JIP curve complementary to F_M_, and reflects the capacity of the intersystem electron carriers and the PSI end acceptors pools to take electrons until full reduction of Q_A_, i.e., closure of all active PSII reaction centres. 

**Figure 3:**
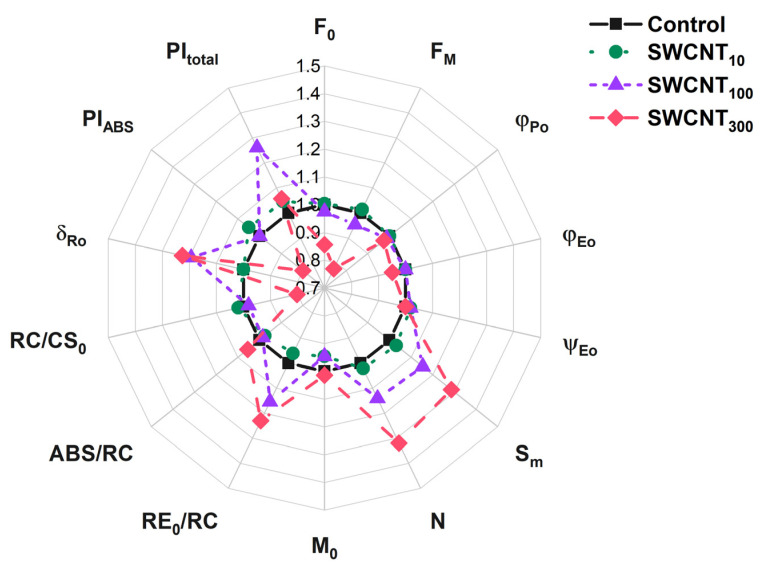
Selected JIP parameters for intact pea leaves treated with distilled water (control), 10, 100 or 300 mg/L of polymer-grafted SWCNT. The JIP parameters values were normalized to those of the control.

Analysis of the electron transfer quantum yields and probabilities revealed that the electron transfer beyond Q_A_^-^ to the intersystem electron carriers was not significantly affected by the applied treatment (parameters φ_Eo_ and ψ_Eo_, Table 1, Figure 3).

The maximum quantum yield of PSII, φ_Po_, was slightly decreased (by 3% on average) only in the variant treated with SWCNT_300_. However, the probability of transfer of an electron from the intersystem electron carriers to the terminal PSI acceptors, δ_Ro_, and the electron flux towards the PSI end acceptors, RE_0_/RC, both were enhanced significantly by about 20% in SWCNT_100_ and SWCNT_300_ (Figure 3). 

Although the maximum quantum yield of PSII primary photochemical reaction was only slightly decreased, the PI_ABS_ parameter, reflecting the performance of PSII absorbed energy conservation as reduced intersystem electron carriers, was lower by about 15% in SWCNT_300_ relative to the control. Interestingly, the overall performance of PSI, PSII and the intersystem electron transport, PI_total_, was not significantly affected. Although the RC/CS_0_ parameter evidenced for 20% decrease in the number of active PSII reaction centres, this change was not paralleled by increase in the absorption per active reaction centre (ABS/RC Table 1, Figure 3). 

### 2.4. PSI Activity 

**Figure 4:**
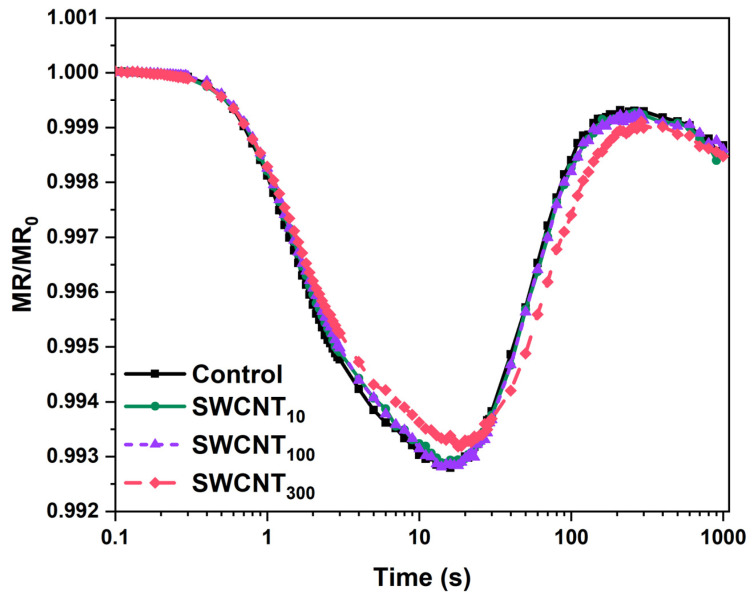
Curves of the modulated reflection (MR) at 820 nm (relative to MR_0_ and presented on a semi-logarithmic scale) recorded for intact pea leaves sprayed with distilled water (control), 10, 100 or 300 mg/L SWCNT.

**Figure 5:**
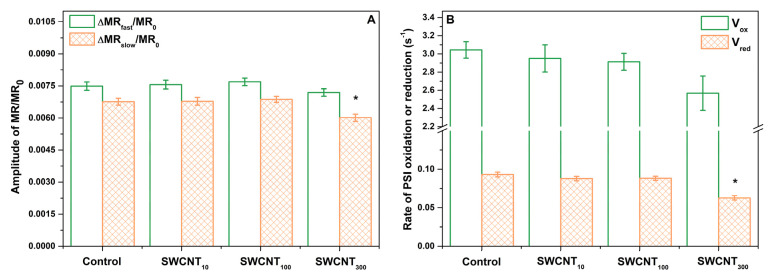
Relative amplitudes of initial decrease (ΔMR_fast_) and subsequent rise (ΔMR_slow_) of the MR signal (**A**) and rates of P_700_ oxidation and re-reduction (**B**). Asterisks indicate statistically significant differences to the respective control revealed with ANOVA and Holm-Sidak ad hoc test at α = 0.05.

As the JIP test performed on SWCNT_100_ and SWCNT_300_ samples indicated higher electron flux towards the PSI acceptor side, we further analysed the modulated reflection at 820 nm for more detailed inspection of the function of PSI. Adopting approach similar to the one applied by Guo et al. [25] we examined the relative MR transients (Figure 4) by calculating the amplitude of MR signal changes (Figure 5A) and the rate of P_700_ oxidation (V_ox_) and re-reduction (V_red_, Figure 5B). 

The MR transients recorded during illumination with actinic light showed characteristic shape including initial fast decrease of the MR signal (ΔMR_fast_) from MR_0_ to a minimal level reached at about 10 ms, that was followed by slower increase in MR intensity (ΔMR_slow_) reaching a plateau at 100 ms (Figure 4). The decrease of the MR at 820 nm (and respectively–increase in absorption at 820 nm) is accompanied by lowering of the absorption at 700 nm i.e., photobleaching at 700 nm, which in turn correlates with enhancement of primary P_700_ and PC oxidation [26]. Thus, this MR phase is governed by the primary photochemical activity of PSI. The following increase of MR reflects the gradual re-reduction of P_700_^+^ and PC^+^ by electrons donated by the intersystem electron carriers, leaving this phase strongly dependent on the PSII function [26]. Therefore, at the point of the minimal MR the rates of P_700_ and PC oxidation and re-reduction are equal.

In all the tested variants, the amplitude of ΔMR_fast_ was higher than the amplitude of ΔMR_slow_ indicating that PSI does not reach full re-reduction after the act of its primary oxidation. The SWCNT treated plants did not show any variation from the control in that respect. 

Moreover, neither the amplitudes of MR, nor the rates of oxidation and re-reduction of P_700_ and PC were influenced by the treatment with P85 only (Figure S1B).

While, the amplitude of ΔMR_fast_ decrease did not change significantly upon SWCNT treatment in any of the tested concentrations, we found significant decrease in the extent of re-reduction of P_700_^+^ and PC^+^ as indicated by the smaller ΔMR_slow_ rise in SWCNT_300_ (Figure 5A). Similarly, while V_ox_ remained unchanged in the SWCN-treated plants, the rate of re-reduction, V_red_ (being highly dependent on PSII activity and the redox poise of the intersystem electron carriers), decreased by about 30% in SWCNT_300_ (Figure 5B). The amplitudes of MR signal variation and the rates of P_700_ (and PC) oxidation and re-reduction were not significantly changed by SWCNT_10_ and SWCNT_100_ (Figure 5).

## 3. Discussion

The complementary pieces of information obtained through analyses of both Chl fluorescence induction curves and the modulated reflection at 820 nm allowed for comprehensive examination of the operation of the donor side of PSII, the acceptor side of PSI and the intersystem electron transport in pea plants treated with different concentrations of polymer-modified SWCNT and polymer only.

The polymer and the lowest tested concentration of SWCNT–10 mg/L did not appear to cause any changes in the functionality of both PSI and PSII based on the analyses of the fluorescence induction curves intensity and shape (Figure 1 and Figure 2, Figure S1), JIP test (Table 1, Figure 3) and the reflection at 820 nm (Figure 4 and Figure 5). In all of the applied analyses, SWCNT_100_ exhibited intermediary values between SWCNT_10_ and SWCNT_300_ following the same tendency as the highest tested concentration and strongly suggesting concentration-dependent effect.

Foliar spraying with carbon-based nanomaterials, especially when in high concentrations, may leave dark spots on leaf surface as illustrated by IMAGING-PAM, (Figure S2). SWCNT on the leaf lamina might decrease the intensity of measuring light reaching the photosynthetic apparatus or re-absorb part of the Chl fluorescence thus affecting the objectivity of Chl fluorescence analyses. In an attempt to assess the extent to which these effects bias our results, we used IMAGING-PAM to compare the mean values of selected fluorescence parameters in the whole leaf area with those in dark spot-free regions in the SWCNT_300_ variant (Figure S3). Indeed, F_0_ and F_M_ in dark spot-free leaf regions were higher than in whole leaf area by less than 10%, and no difference in F_V_/F_M_ ratio was observed (Figure S3). Thus, the unspecific effects of the SWCNT presence on the leaf surface (i.e., re-absorption and/or shading) might be accounted for about 10% of the detected changes in Chl fluorescence intensity. Importantly, data provided by OJIP curves, recorded on the leaf area consisting of both dark spots and spot-free regions, demonstrate more substantial differences in F_0_ and F_M_ when control and SWCNT_300_ plants are compared, F_0_ and F_M_ for SWCNT_300_ being lower by 15% and 23%, respectively (Table 1). Hence, these changes are not only due to the altered optical leaf properties.

We found that in SWCNT_300_ samples the functionality of the donor side of PSII was unaffected with no significant changes within the initial 2 ms of the fluorescence induction curve, i.e., the function of the oxygen evolving complex was preserved [23]. Also, according to the JIP test parameters M_0_, φ_Eo_ and ψ_Eo_ (Table 1), no changes were found in the rate of PSII reaction centres closure and the efficiency of electron transfer to intersystem electron carriers. The functionality of PSII as judged by φ_Po_ was slightly but significantly inhibited by the SWCNT treatment (Table 1). Next, the concentration-dependent slowdown of fluorescence rises in the J-I phase (Figure 2A) indicated retarded reduction of the PQ pool [21]. The negative bands at the I-P phase differential curves (Figure 2B) reflect impeded reduction of the terminal electron acceptors of PSI. When considering the negative bands at the J-I and I-P phases it should be taken into account that fluorescence rise is affected both by the donor and the acceptor sides, i.e., by decrease of the number of active PSII reaction centres and/or altered activity of PSI. Indeed, we observed decrease in the density of active PSII centres (RC/CS_0_, Table 1) which means a smaller electron flux towards the PQ pool leading to slower rise of fluorescence. This effect might be due to increase in the reactive oxygen species formation as reported previously for SWCNT-treated plants [8,10]. The increased flux of electrons towards PSI end electron acceptors (RE_0_/RC) and the higher probability for transfer of an electron from the intersystem electron carriers to PSI acceptor side (δ_Ro_, Table 1) indicate that the fluorescence rise slowdown at the J-I and I-P phases might be also caused by enhanced PSI activity. Counterintuitively, neither the initial drop in the amplitude of the MR signal (ΔMR_fast_, Figure 5A), nor the rate of initial P_700_ oxidation (V_ox_, Figure 5B), which are defined by the PSI functionality solely, were affected. However, the ΔMR_slow_ and V_red_ were significantly lower in SWCNT_300_ relative to the control (Figure 5). Schansker et al. [22] argued that the recovery of I-P phase in the process of light to dark adaptation occurs simultaneously with recovery of the MR slow rise. Similarly, Strasser et al. [17] ascribed the gradual decrease of the amplitude of MR rise, which was accompanied by disappearance of the I phase in desiccated plants, to inhibition of the intersystem electron transport from PQH_2_ to PC. A rough parallel can be drawn between the effects of SWCNT_300_ concentration and the data presented by Guo et al. and Schansker et al. [25,27] regarding the action of dibromothymoquinone (DBMIB, acting as a Cyt *b_6_f* inhibitor preventing electron transfer from PQH_2_ to PC) and methylviologen (MV, pulling electrons from P_700_ ahead of ferredoxin-NADP reductase). Both DBMIB and MV caused appearance of negative I-P bands and decrease in both V_ox_ and V_red_ which was more strongly expressed for V_red_ [25]. The action of MV was related to strongly lowered or no increase of MR signal reflecting P_700_ (and PC) re-reduction which was explained by the fact that MV pulls electrons from P_700_ thus not allowing electrons to accumulate in the PQ pool and to re-reduce P_700_ [25,27]. Hence, by complementing prompt Chl fluorescence with MR data, it can be hypothesized that in the SWCNT_300_-treated plants the slower fluorescence rise in the I-P and J-I phases is at least partially due to increased electron flow respectively (i) at the acceptor side of PSI and (ii) at the level of PQH_2_, and/or at the level of Cyt *b_6_f*. The latter hypothesis should be taken with caution due to the significant deactivation of Q_A_ reducing PSII reaction centres (Table 1, Figure 3). The significant increase of S_m_ and N parameters of the JIP test (Table 1, Figure 3) corroborates with those hypotheses as they indicate for larger pool of electron carriers, which have to be reduced before full reduction of all Q_A_ in all the active RC. Interestingly, the increase of S_m_ and N is not accompanied by changes in the probability of electron transport from Q_A_^-^ towards PQ (parameter ψ_Eo_, Table 1). This effect might be due to decreased number and photochemical activity of the PSII reaction centres (RC/CS_0_, φ_Po_, Table 1) or possibly due to donation of electrons to SWCNT only at the level of PQ and beyond (reflected after the J point of the OJIP curves). The retarded fluorescence rise in the J-I and I-P phases might be due to downregulation of electron transport through Cyt *b_6_f* known to be a subject of ‘photosynthetic control’ by thylakoid lumen acidity [28]. Our recent study demonstrates that the proton motive force in SWCNT_300_ treated plants was slightly lower than in control plants [12] indicating that the slower increase of Chl fluorescence in the J-P phase is not due to Cyt *b_6_f* control. It is still to be cleared out if SWCNT are capable of pulling electrons at the PSI or Cyt *b_6_f* level or the observed phenomenon represents indirect effect of the SWCNT treatment.

In concert with our results, it was earlier suggested that PSI is capable of electron donation to SWCNT when the two are physically interacting [5,6]. Thus, it appears highly possible that the agent extracting electrons from the photosynthetic electron transport chain is SWCNT. Moreover, SWCNT were found to be capable of reaching and entering the chloroplasts in *Arabidopsis* [4,8]. It should be noted that in these experiments SWCNT were either applied on a leaf surface with compromised epidermis or infiltrated into the leaves, thus omitting the barrier properties of cuticular waxes. The results of our recent work, however, suggest penetration of SWCNT into chloroplasts upon non-invasive foliar application of polymer-grafted SWCNT [12] which was also applied in this work. 

The contribution of CEF to the observed SWCNT-induced effect should be taken into account as well. Short-term heat stress leads to substantial enhancement of the NAD(P)H dehydrogenase-dependent CEF and it was argued that this response improves heat stress resistance of some rice lines [29]. Zhou et al. [30] reported that maize plants resistant to drought stress differ from the susceptible ones by their capability to retain the operation of CEF. Since SWCNT can be viewed as a novel and unexplored abiotic stress factor, it cannot be ruled out that the lower V_red_ and ΔMR_slow_ in SWCNT_300_ reflects activation of CEF. Our recent work illustrates that foliar application of SWCNT alters CO_2_ assimilation [12], which might lead to over-reduction of the photosynthetic electron transport chain and activation of CEF as a safety valve. 

Recent investigation of the photosynthetic activity of *Arabidopsis* and maize plants treated with SWCNT evidenced for increased photosynthetic electron transport [4,10]. Giraldo et al. [4] ascribed these results to increased light absorption properties of the photosynthetic apparatus due to the wider absorption spectrum of SWCNT, which were argued to transfer excitons to the photosynthetic apparatus. Contrary to these observations, in our experimental conditions we did not find significantly enhanced overall photosynthetic activity (as judged by the PI_total_ parameter, Table 1), probably due to differences in the type, surface modification and method of application of SWCNT utilized. It should be noted that the instrumentation utilized in the current work employs excitation light with wavelength strongly limited to 650 nm. Thus, conclusions regarding the question of complementation of the absorption spectra of the photosynthetic complexes with SWCNT are not relevant.

## 4. Materials and Methods

### 4.1. SWCNT Preparation

SWCNT were purchased from Sigma-Aldrich (>77% carbon as SWCNT; diameter: 0.7–1.1 nm, length: 300–2300 nm). With the aim of achieving stable dispersion of the water suspensions of SWCNT, the nanotubes were grafted with poly(ethylene oxide)_26_-block-poly(propylene oxide)_40_-block-poly(ethylene oxide)_26_ triblock copolymer (“Pluronic” P85, from BASF) as in a procedure described in [19,31]. In brief, deionized water solution of the triblock P85 co-polymer and SWCNT were mixed during sonication and further diluted with deionized water to 10, 100 and 300 mg SWCNT/L (denoted as SWCNT_10_, SWCNT_100_, SWCNT_300_). Immediately prior to treatment SWCNT suspensions were sonicated for 30 min. 

### 4.2. Plant Material

7-day-old pea plantlets (*Pisum sativum* cv. RAN1) with two fully developed leaf pairs, grown in the same conditions as in [32] were sprayed with around 3 mL/plant of SWCNT at concentrations of 10, 100 or 300 mg/L. Chl fluorescence and modulated reflection at 820 nm measurements of intact pea leaves were performed 7 days after the SWCNT-treatment. Control plants were sprayed with distilled water. In addition, to check for specific effects due to P85 co-polymer, we examined pea plants sprayed with P85 solutions with concentrations corresponding to those used for SWCNT dispersion preparations.

### 4.3. Photosynthetic Performance

Fast kinetics of prompt Chl *a* fluorescence and modulated reflection at 820 nm of dark adapted (for 30 min) plants were recorded simultaneously within 0.5 s with M-PEA fluorimeter (Hansatech Instruments Ltd., King’s Lynn, UK). 

### 4.4. Analysis of Chl Fluorescence Induction Curves

Prompt Chl fluorescence induction curves were obtained upon application of high-intensity 3000 µmol photons m^−2^ s^−1^ light pulse at 650 ± 10 nm wavelength. JIP analysis of the fluorescence induction curves was done according to Strasser et al. [20] and Goltsev et al. [14]. The formulae used for calculation of selected JIP parameters examined in the current work are presented in Table S1. Variable Chl fluorescence curves were obtained by double normalization according to the formula:

$$V_{t}=\left(F_{t}-F_{0}\right)/\left(F_{P}-F_{0}\right)$$

where F_t_ is the fluorescence intensity at time t after starting the measuring protocol, F_0_ is the minimal fluorescence recorded at 20 µs and F_P_ is the maximal fluorescence intensity (at around 300 ms, also known as F_M_). Differential curves of three complementary parts of the Chl fluorescence transients were constructed by subtraction of the respective double normalized curves, recorded for the control, from the curves of each SWCNT- or P85-treated sample:

$${\Delta W}_{\mathrm{OJ}}{=W}_{\mathrm{OJ}}^{\mathrm{treated}}{-W}_{\mathrm{OJ}}^{\mathrm{control}}$$

$${\Delta W}_{\mathrm{JI}}{=W}_{\mathrm{JI}}^{\mathrm{treated}}{-W}_{\mathrm{JI}}^{\mathrm{control}}$$

$${\Delta W}_{\mathrm{IP}}{=W}_{\mathrm{IP}}^{\mathrm{treated}}{-W}_{\mathrm{IP}}^{\mathrm{control}}$$

where:

$$W_{\mathrm{OJ}}=\frac{F_{t}-F_{0}}{F_{J}-F_{0}}$$

$$W_{\mathrm{JI}}=\frac{F_{t}-F_{J}}{F_{I}-F_{J}}$$

$$W_{\mathrm{IP}}=\frac{F_{t}-F_{I}}{F_{P}-F_{I}}$$

F_J_ being fluorescence intensity at 2 ms, and F_I_—fluorescence intensity at 30 ms. 

### 4.5. Analysis of Modulated Reflection at 820 nm

For modulated reflection (MR) measurements, a LED light at 820 ± 25 nm and 100% intensity was applied. MR values were presented relative to the initial reflection at 820 nm (MR_0_): MR/MR_0_. The amplitude of the fast drop of MR signal was defined as:

$${\Delta \mathrm{MR}}_{\mathrm{fast}}{=\mathrm{MR}}_{0}{-\mathrm{MR}}_{\min}$$

and the amplitude of the slow rise of MR signal was calculated by the formula:

$${\Delta \mathrm{MR}}_{\mathrm{slow}}{=\mathrm{MR}}_{\mathrm{terminal}}{-\mathrm{MR}}_{\min}$$

where MR_0_ is the average reflection registered between 0.02 and 0.07 ms after beginning of measurement, MR_min_ is the lowest MR value reached after initial fast decrease of MR signal and MR_terminal_ is the averaged MR signal within 270 to 400 ms of the measurement protocol. The slopes of the initial decrease and subsequent increase of the MR signal, representing the apparent rates of P_700_ oxidation and re-reduction, were calculated by linear regression analysis in the time ranges 0.6–1.1 ms and 30–70 ms, respectively.

### 4.6. Statistical Analysis

One-way ANOVA with α = 0.05 and Holm-Sidak ad hoc test was performed in OriginLab 2018. The summarized results (averaged values with standard errors) represent data obtained in 3 independent experiments each involving 10 measurements per variant.

## 5. Conclusions

**Figure 6:**
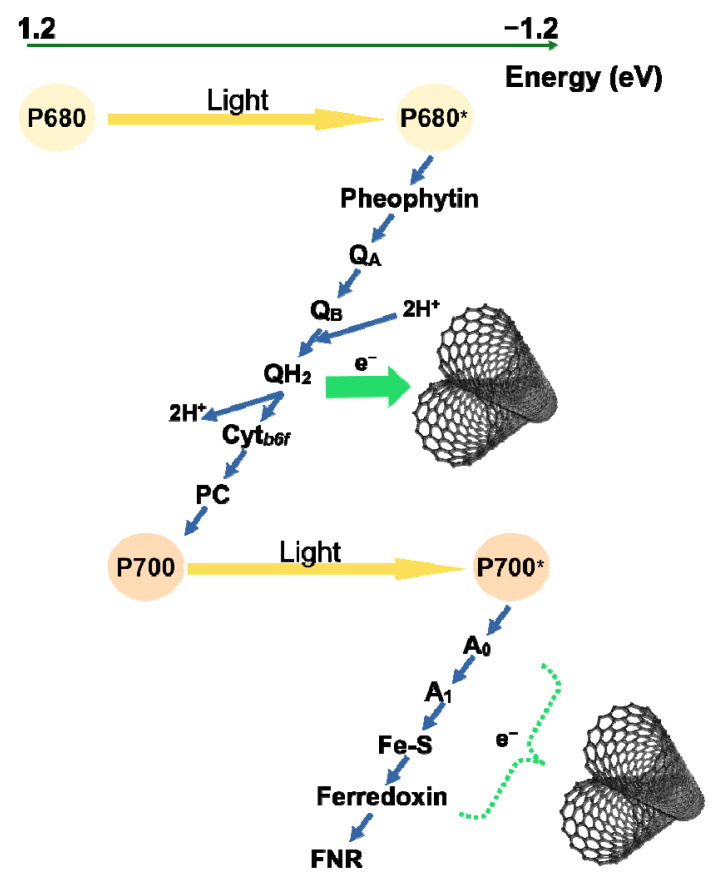
Proposed mechanism of interaction between the photosynthetic electron transport chain of higher plants and P85 polymer-modified SWCNT. SWCNT are capable to divert electrons from the photosynthetic electron transport chain at the level of plastoquinone pool and/or photosystem I end acceptors in vivo.

The specific physical properties of carbon-based nanotubes offer various possibilities for their application in agriculture and biotechnology. This requires in-depth knowledge of possible interactions with the photosynthetic apparatus. Here we have focused on the possible interactions between SWCNT and the components of electron transport chain. Although SWCNT did not affect the donor side of PSII, they caused decrease in the number of active PSII centres. Our data suggest that foliar application of polymer-grafted SWCNT affects the higher plant photosynthetic apparatus at several levels. SWCNT slowed down the electron transfer by the intersystem electron carriers and lowered the extent and rate of reduction of the end acceptors of PSI. These observations provide the first in vivo indication for possible electron transfer between the photosynthetic electron-transport chain (the end acceptors of PSI and PQH_2_) and SWCNT, thus, supporting our hypothesis on interaction between the SWCNT and photosynthetic electron transport chain (Figure 6). However, further research is needed to unequivocally prove if the observed effects are due to specific interaction between SWCNT and the photosynthetic apparatus.

## Figures and Tables

**Table 1:** Mean values of selected JIP test parameters for intact pea leaves treated with 10, 100 or 300 mg/L polymer-grafted SWCNT.

	Control	SWCNT_10_	SWCNT_100_	SWCNT_300_
**F_0_**	9231 ± 109	9267 ± 794	9000 ± 542	7900 ± 507 *
**F_M_**	40108 ± 599	40671 ± 360	38262 ± 269	31169 ± 2711 *
φ_Po_	0.77 ± 0.00	0.77 ± 0.02	0.76 ± 0.01	**0.75 ± 0.01** *
**φ_Eo_**	0.41 ± 0.03	0.41 ± 0.01	0.41 ± 0.01	0.39 ± 0.01
**ψ_Eo_**	0.53 ± 0.04	0.54 ± 0.01	0.54 ± 0.02	0.53 ± 0.02
**S_m_**	18.9 ± 1.3	19.5 ± 1.7	21.8 ± 1.6	24.3 ± 1.7 *
**N**	29.1 ± 0.1	29.7 ± 3.5	33.2 ± 3.3	38.4 ± 1.4 *
**M_0_**	0.74 ± 0.11	0.7 ± 0.03	0.7 ± 0.05	0.75 ± 0.05
**RE_0_/RC**	0.26 ± 0.01	0.25 ± 0.02	0.3 ± 0.03 *	0.32 ± 0.01 *
**ABS/RC**	2.02 ± 0.13	1.97 ± 0.12	1.99 ± 0.12	2.13 ± 0.07
**RC/CS_0_**	4608 ± 235	4698 ± 191	4520 ± 166	3695 ± 124 *
**δ_Ro_**	0.31 ± 0.02	0.31 ± 0.01	0.37 ± 0.02 *	0.38 ± 0.01 *
**PI_ABS_**	2.0 ± 0.4	2.1 ± 0.3	2.0 ± 0.3	1.6 ± 0.1
**PI_total_**	0.88 ± 0.12	0.92 ± 0.09	1.11 ± 0.06	0.93 ± 0.06

Asterisks indicate statistically significant differences to the respective control revealed with ANOVA and Holm-Sidak ad hoc test at α = 0.05.

## Data Availability

Data are contained within the manuscript and the Supplementary Materials.

## Supplementary Materials

The following are available online at https://www.mdpi.com/article/10.3390/molecules26195958/s1. Figure S1: Prompt fluorescence induction curves (A) and modulated reflection at 820 nm (B) of intact pea leaves sprayed with distilled water or polymer P85 only at concentrations of 10, 100 and 300 mg/L. Figure S2. Representative chlorophyll fluorescence images of Φ_PSII_ in pea plants sprayed with H_2_O, polymer (10 mg/L, P–10; 100 mg/L, P–100; and 300 mg/L, P–300) and SWCNT (10 mg/L, SWCNT–10; 100 mg/L, SWCNT–100; and 300 mg/L, SWCNT–300). Figure S3. Selected Chl fluorescence parameters measured in pea leaves sprayed with 300 mg/L SWCNT. Analysis was performed on whole leaf area as well as on selected leaf area free of dark spots. Table S1. Definition of selected JIP test parameters.

## Sample Availability

Samples of the compounds are not available from the authors.
