# Supplementary material for: Polymer-Modified Single-Walled Carbon Nanotubes Affect Photosystem II Photochemistry, Intersystem Electron Transport Carriers and Photosystem I End Acceptors in Pea Plants
Source: Molecules. 2021 Oct 1;26(19):5958. doi: 10.3390/molecules26195958 (PMC8512794; doi:10.3390/molecules26195958)
Supplement: Supplementary file 1 [file molecules-26-05958-s001.zip › molecules-1368326-supplementary.pdf]

Supplementary material

Original research manuscript submitted to MDPI Molecules for Special Issue "*Photosystem II Photochemistry in Biotic and Abiotic Stress*"

Title: Polymer-modified single-walled carbon nanotubes affect photosystem II photochemistry, intersystem electron transport carriers and photosystem I end acceptors in pea plants

Authors: Nia Petrova[1,‡,*], Momchil Paunov[2], Petar Petrov[3], Violeta Velikova[4*], Vasilij Goltsev[2], Sashka Krumova[1]

[1] Institute of Biophysics and Biomedical Engineering, Bulgarian Academy of Sciences, Acad. Georgi Bonchev Str., Bl. 21, Sofia 1113, Bulgaria
[2] Faculty of Biology, Sofia University 'St. Kliment Ohridski', 8 Dragan Tsankov Blvd., Sofia 1164, Bulgaria
[3] Institute of Polymers, Bulgarian Academy of Sciences, Akad. G. Bonchev Str., Bl. 103-A, Sofia 1113, Bulgaria
[4] Institute of Plant Physiology and Genetics, Bulgarian Academy of Sciences, Acad. G. Bonchev Str., Bl. 21, Sofia 1113, Bulgaria
‡ current address: Laboratory for Molecular Photobioenergetics, Institute of Plant Biology, Biological Research Centre, Temesvári krt. 62. H-6726 Szeged, Hungary

* Correspondence: zlatkova.nia@gmail.com (N.P.); violet@bio21.bas.bg (V.V.)

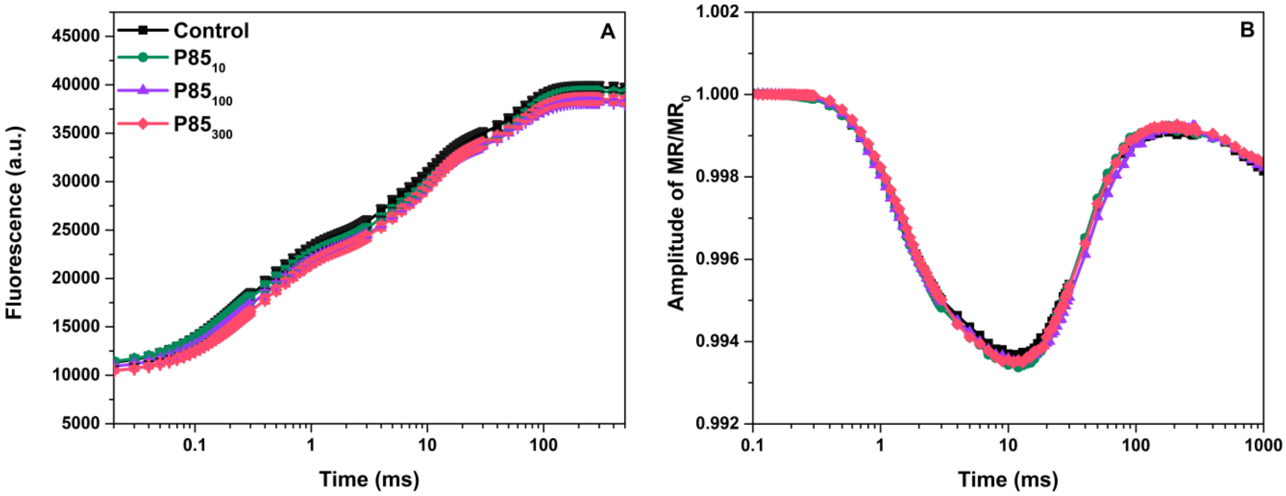

Figure S1. Prompt fluorescence induction curves (A) and modulated reflection at 820 nm (B) of intact pea leaves sprayed with distilled water or polymer P85 only at concentrations of 10, 100 and 300 mg/L.

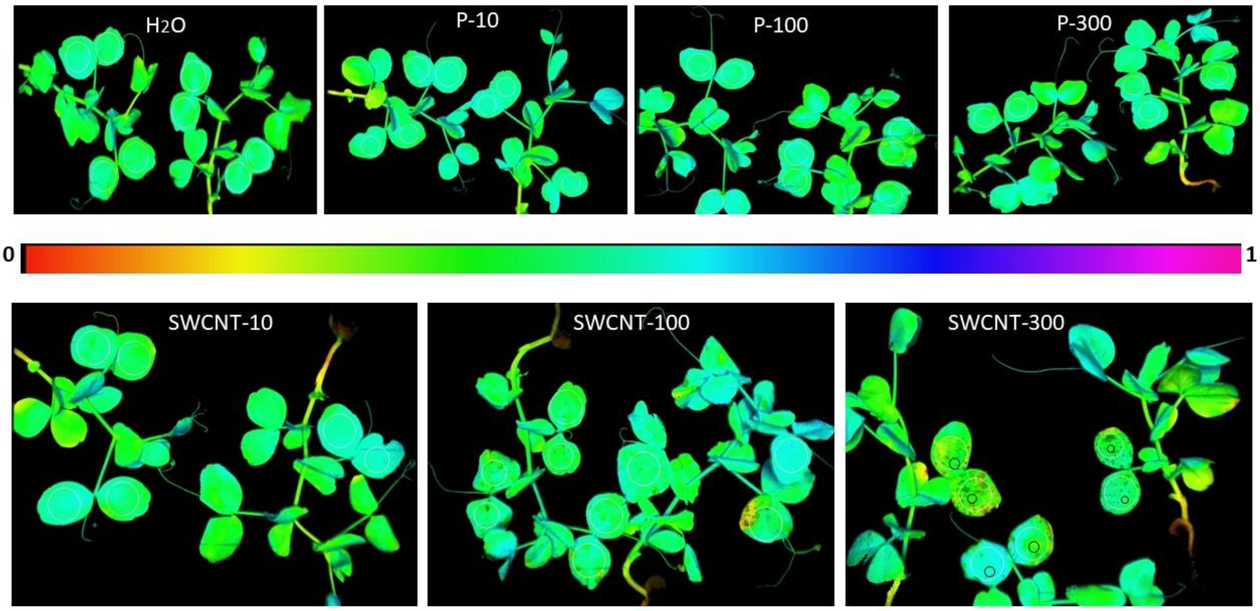

Figure S2. Representative chlorophyll fluorescence images of Φ_{PSII} in pea plants sprayed with H_2O, polymer (10 mg/L, P-10; 100 mg/L, P-100; and 300 mg/L, P-300) and SWCNT (10 mg/L, SWCNT -10; 100 mg/L, SWCNT-100; and 300 mg/L, SWCNT-300). The false colour code depicted ranges from 0 (black) to 1 (pink) in fluorescence intensity. The images were recorded by means of IMAGING-PAM MAXI (Walz, Germany) instrument as in Velikova et al. [12].

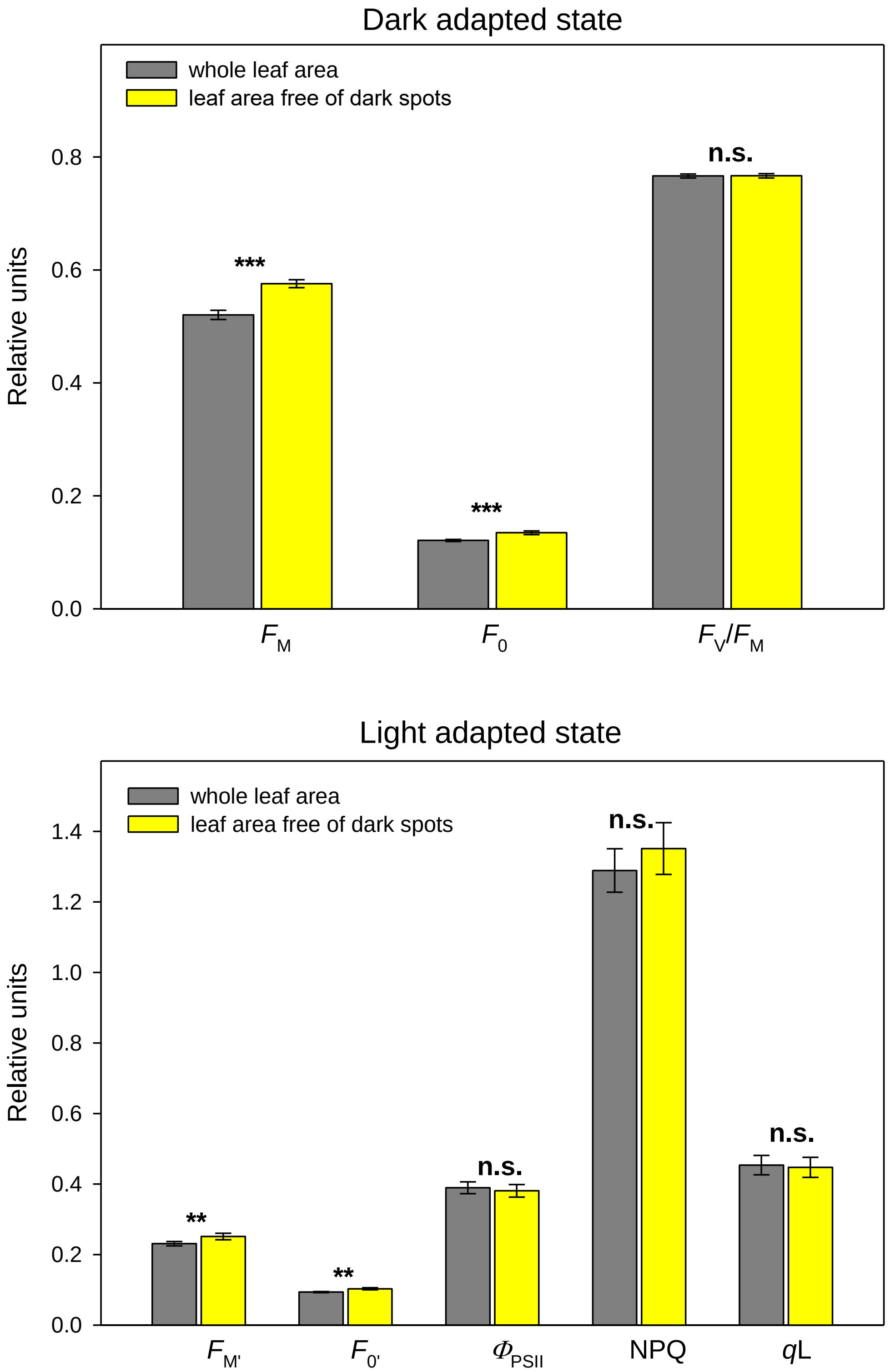

Figure S3. Maximum (F_M, $F_{M'}$) and minimum (F_0, $F_{0'}$) fluorescence measured in dark and light adapted state, maximum quantum yield of photosystem II (F_V/F_M), quantum efficiency of photosystem II photochemistry (Φ_{PSII}), non-photochemical quenching (NPQ) and redox state of photosystem II (qL) in pea leaves sprayed with 300 mg/L SWCNT. Chlorophyll fluorescence parameters were measured by IMAGING-PAM MAXI (Walz, Germany) instrument as in Velikova et al. [12]. Analysis was performed on whole leaf area (dark gray bars) and on selected leaf area free of dark spots (yellow bars) as indicated in **Figure S2** (last panel).

Table S1. Definition of selected JIP test parameters [14, 20].

JIP parameter	Interpretation	Mathematical equation
F_0	Minimal fluorescence	$F_{20\mu s}$, fluorescence recorded at t=20 μs after the start of measurement
F_M	Maximal fluorescence	F_P, fluorescence at the P-step of the fluorescence induction curve
φ_{Po}	Maximal quantum yield of the primary photochemical reaction in PSII in dark-adapted samples	$\frac{F_M - F_0}{F_M}$
φ_{Eo}	Quantum yield of the process of electron transfer from Q_A^- to electron carriers beyond Q_A^-	$\varphi_{Po}(1 - V_J)$ where V_J is the variable chlorophyll fluorescence at phase J of the fluorescence induction curve: $V_J = \frac{F_J - F_0}{F_M - F_0}$
ψ_{Eo}	The probability that the energy of an exciton trapped by active PSII reaction centre (RC) will be utilized for electron transport beyond Q_A^-	$1 - V_J$
S_m	Total electron carriers, per RC, reduced during the time of the induction rise (from F_O to F_M)	$\frac{Area}{F_M - F_0}$, where Area is the total complementary area between the fluorescence induction curve and F = F_P
N	Turnover number, expressing how many times Q_A is reduced until F_M is reached	$S_m M_0 \left(\frac{1}{V_J}\right)$
M_0	Approximated initial slope (in ms^{-1}) of the fluorescence transient normalized to the maximal variable fluorescence. Reflects the maximal rate of initial Q_A reduction	$\frac{4(F_{300\mu s} - F_{50\mu s})}{F_M - F_0}$, where $F_{300\mu s}$ and $F_{50\mu s}$ are fluorescence intensities at 300 μs and 50 μs after beginning of measurement
RE_0/RC	Flux of electrons reaching the end carriers at the acceptor side of PSI as per RC	$M_0\left(\frac{1}{V_J}\right)(1 - V_I)$, where V_I is the variable chlorophyll fluorescence at phase I of the JIP transient: $V_I = \frac{F_I - F_0}{F_M - F_0}$
ABS/RC	Absorption flux (exciting PSII antenna Chl *a* molecules) per RC	$M_0\left(\frac{1}{V_J}\right)\left(\frac{1}{\varphi_{Po}}\right)$
RC/CS_0	Number of active (Q_A reducing) PSII reaction centres per cross section	$\varphi_{Po} F_0\, V_J / M_0$
δ_{Ro}	Probability with which an electron from the intersystem electron carriers is transferred to reduce end electron acceptors at the PSI acceptor side	$\frac{1 - V_I}{1 - V_J}$
PI_{ABS}	Performance index for energy conservation from photons absorbed by PSII until the reduction of intersystem electron acceptors	$\frac{RC}{ABS} \frac{\varphi_{Po}}{1 - \varphi_{Po}} \frac{\psi_{Eo}}{1 - \psi_{Eo}}$
PI_{total}	Performance index for energy conservation from photons absorbed by PSII until the reduction of PSI end electron acceptors	$PI_{ABS} \frac{\delta_{Ro}}{1 - \delta_{Ro}}$
